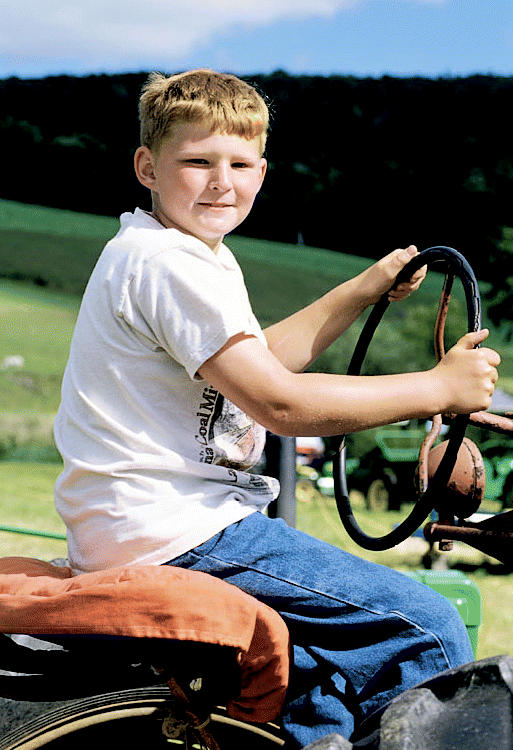# Agriculture: Farm Chore Checkup

**DOI:** 10.1289/ehp.112-a804b

**Published:** 2004-10

**Authors:** Julia R. Barrett

Nearly 23,000 children are hurt doing farmwork in the United States each year, and approximately 100 children are killed, according to the National Agricultural Statistics Service. To combat this problem, the National Children’s Center for Rural and Agricultural Health and Safety in Marshfield, Wisconsin, developed the North American Guidelines for Children’s Agricultural Tasks (NAGCAT) in the late 1990s to help farm parents assess whether their children aged 7–16 are developmentally ready to safely complete various farm tasks. A recent study now offers evidence that NAGCAT has been effective at preventing injuries to children working on farms.

The study was led by pediatric scientist Anne Gadomski of the Bassett Research Institute in Cooperstown, New York, and published in the October 2004 *American Journal of Public Health*. It is the first randomized, controlled trial to test the efficacy of these guidelines in preventing farm injuries.

William Pickett, a researcher on agricultural injury at Queens University in Kingston, Ontario, says many interventions are directed at children, which may not be an effective strategy. “You can have the most highly educated, most informed child around,” he says, “but they are not necessarily the ones making the decisions about what they do on the farm and where they are allowed to go.”

NAGCAT, on the other hand, provides guidance for those who do make such decisions. The guidelines are conveyed through a professional resource manual and parent booklets. Each booklet covers a set of related farm tasks, such as animal care or tractor work, using a poster format to describe the task, adult responsibilities, potential hazards, and necessary safety precautions. The posters also list developmental abilities a child must possess to perform the task.

The study involved 2,454 children on 845 farms in central New York. Some of the farms received NAGCAT information, while others did not. Over 21 months, the researchers collected data on children’s injuries, what they were doing when injured, their general responsibilities, and the number of hours worked.

Although the two groups did not differ significantly in overall injury incidence, farms that received NAGCAT information reported fewer injuries related to tasks described in the guidelines. Gadomski says, “We suspect that the average age of the child is going up in terms of being assigned certain tasks now that the parent has a guideline to help them make that assessment. The other issue is [parents now have] some idea of how much supervision certain aged children require in order to do the job safely.” For the most part, children under 7 are not ready to engage in productive agricultural work, says Nancy Esser, an agricultural youth safety specialist at the center. The center therefore recommends that young children not be involved in such work.

The study is a welcome addition to the literature in an area where there are few published trials, says Pickett. But NAGCAT addresses only one portion of the pediatric farm injury problem, he says. Similar efforts are needed to address injuries that occur among young children—not necessarily workers—who accompany their worker parents into the farm environment.

## Figures and Tables

**Figure:**